# The Role of Diverse Immune Cells in Sarcoidosis

**DOI:** 10.3389/fimmu.2021.788502

**Published:** 2021-11-19

**Authors:** Hui Zhang, Ulrich Costabel, Huaping Dai

**Affiliations:** ^1^ Department of Pulmonary and Critical Care Medicine, Center of Respiratory Medicine, China-Japan Friendship Hospital, Peking Union Medical College, Beijing, China; ^2^ Center for Interstitial and Rare Lung Diseases, Pneumology Department, Ruhrlandklinik, University Hospital, Essen, Germany; ^3^ Department of Pulmonary and Critical Care Medicine, Center of Respiratory Medicine, China-Japan Friendship Hospital, Beijing, China; ^4^ National Center for Respiratory Medicine, Beijing, China; ^5^ Institute of Respiratory Medicine, Chinese Academy of Medical Sciences, Beijing, China; ^6^ National Clinical Research Center for Respiratory Diseases, Beijing, China

**Keywords:** sarcoidosis, Th17 cells, regulatory T cells, macrophages, B cells, dendritic cells, natural killer cells

## Abstract

Sarcoidosis is a systemic inflammatory disorder of unknown etiology characterized by tissue infiltration with macrophages and lymphocytes and associated non-caseating granuloma formation. The disease primarily affects the lungs. Patients suffering from sarcoidosis show a wide range of clinical symptoms, natural history and disease outcomes. Originally described as a Th1-driven disease, sarcoidosis involves a complex interplay among diverse immune cells. This review highlights recent advances in the pathogenesis of sarcoidosis, with emphasis on the role of different immune cells. Accumulative evidence suggests Th17 cells, IFN-γ-producing Th17 cells or Th17.1 cells, and regulatory T (Treg) cells play a critical role. However, their specific actions, whether protective or pathogenic, remain to be clarified. Macrophages are also involved in granuloma formation, and M2 polarization may be predictive of fibrosis. Previously neglected cells including B cells, dendritic cells (DCs), natural killer (NK) cells and natural killer T (NKT) cells were studied more recently for their contribution to sarcoid granuloma formation. Despite these advances, the pathogenesis remains incompletely understood, indicating an urgent need for further research to reveal the distinct immunological events in this process, with hope to open up new therapeutic avenues and if possible, to develop preventive measures.

## Introduction

Sarcoidosis is a multisystemic inflammatory disorder of unknown etiology characterized by the presence of non-caseating granulomas. The disease commonly affects the lungs and other organs including eyes, skin, liver, spleen, and lymph nodes (1). The histological features of a sarcoidosis granuloma include well-formed and concentrically arranged layers of immune cells, with a prominent central core of macrophage aggregates, epithelioid cells and multinucleated giant cells, accompanied by lymphocytes, mostly T cells, with a few interposed dendritic cells (DCs) located in an outer layer, and isolated collections of B lymphocytes surrounding the granulomas in some cases (2). In the lung, sarcoid granulomas typically coalesce along the lymphatic routes in the pleura, interlobular septa, and bronchovascular bundles (3).

The diagnosis of sarcoidosis is based on a compatible clinical presentation, together with the finding of non-necrotizing granulomatous inflammation, and the exclusion of alternative causes of granulomatous disease. A new guideline offers suggestions on diagnosis and detection of sarcoidosis for physicians in clinical practice, by using systematic reviews of the evidence to inform clinical recommendations in favor of or against various diagnostic tests in patients with suspected sarcoidosis (2). Emerging biomarkers, including serum biomarkers, genetic biomarkers, imaging biomarkers, and fibrotic biomarkers, such as high level of serum angiotensin converting enzyme (SACE), upregulation of Th1 immune response genes, abnormal PET-CT findings and downregulation of T cell receptor signaling pathways, reflecting the complex interplay among diverse immune cells, may provide evidence supporting or refuting the diagnosis with diverse sensitivity and specificity (4). The incidence, prevalence and disease burden of sarcoidosis vary greatly depending on geographical regions, sexes, ethnicities and age groups (5).

The cause of sarcoidosis remains uncertain, but various factors, including infection, genetic predisposition, and environmental conditions, may play a role (6). Consequently, sarcoidosis has quite different clinical phenotypes, resulting in diverse disease outcomes. Many asymptomatic patients reach remission spontaneously even without treatment. Patients suffering from cough, shortness of breath, chest pain and pronounced fatigue can improve or remain in stable condition receiving appropriate therapy, whereas a minority develops chronic progressive disease accompanied by severe complications, such as pulmonary hypertension and pulmonary fibrosis, impairing the health-related quality of life, or even leading to death (7).

There is no cure for chronic sarcoidosis, and treatment only dampens the granulomatous process and its clinical consequences (8). Systemic corticosteroids remain the standard treatment with many well-known side effects (9, 10). Alternative options include methotrexate, azathioprine, antimalarial drugs and leflunomide, as well as biologicals such as infliximab for chronic and refractory sarcoidosis (11). Symptomatic and supportive measures and keeping close follow-up of patients are also crucial (12).

**Figure 1:**
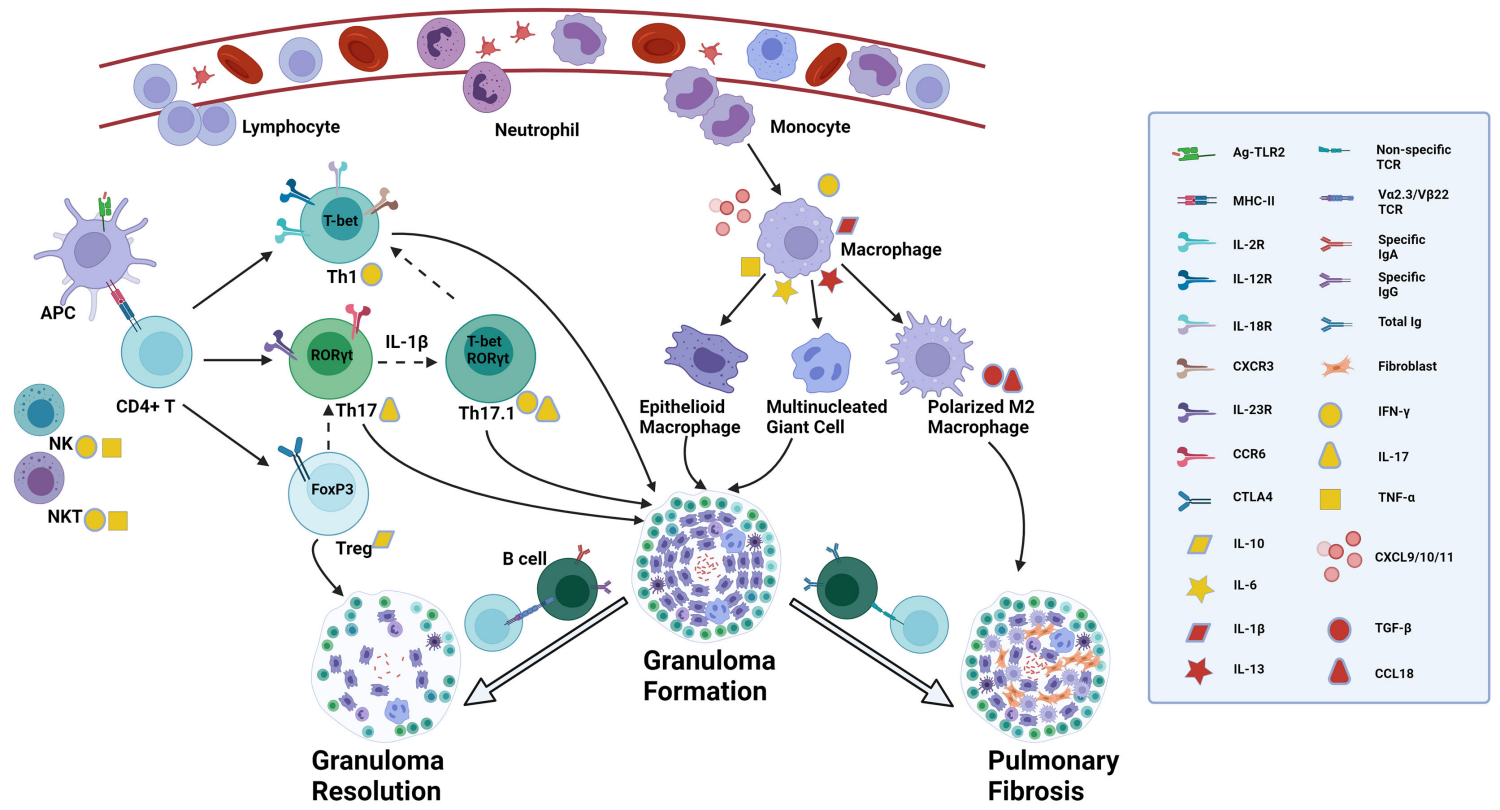
Proposed role of diverse immune cells involved in sarcoidosis. The presence of a still unknown antigen in the lungs triggers antigen recognition through innate immune receptors such as TLR2. DCs and alveolar macrophages, acting as APCs, process and present antigens through MHC II - TCR complex to CD4+ T cells, activated by the surrounding abundant cytokines like TNF-α and IFN-γ secreted by NK cells and NKT cells and by activated macrophages. Activated CD4+ T cells can differentiate into various effector T cells depending on the immune microenvironment. In a Th1 cytokine environment, they convert into Th1 cells which express T-bet mRNA, secrete IFN-γ, and interact with matched ligands through IL-2R, IL-12R, IL-18R and CXCR3. Under Th17 inducing conditions, Th17 cells are the prominent effector T cells which express RORγt mRNA in the nucleus, and IL-23R and CCR6 on the membrane, and produce IL-17. In a suppressive immune milieu, Treg cells expressing FoxP3 mRNA play an immune regulatory role by expressing CTLA4 on the membrane and secreting IL-10. There is a delicate balance of transcription factor expression, indicating T-cell plasticity. Th17 cells can co-express RORγt and T-bet mRNA, converting to the so-called Th17.1 cells, regulated by IL-1β. Th17.1 cells capable of producing simultaneously IL-17 and IFN-γ, may lose the expression of RORγt mRNA and ultimately differentiate into T-bet expressing Th1 cells through uncertain mechanisms, secreting IFN-γ alone. Treg cells can lose the expression of FoxP3 and instead express RORγt, thereby turning into Th17 cells in certain circumstances. Upon the recognition of antigens, activated macrophages secrete diverse chemokines, such as CXCL10, attracting and recruiting neutrophils, monocytes and lymphocytes from blood into the lungs, and CXCL9/11 which is recognized by CXCR3 on Th1 cells and promotes further accumulation of Th1 cells. Activated macrophages also secrete cytokines such as TNF-α, IL-1β, IL-6 and IL-13 which mediate the formation of epithelioid macrophages and multinucleated giant cells. Patients with specific TCR Vα2.3/Vβ22 are able to completely clear antigens by specific IgA and IgG through T-B-cell interaction and the granulomas resolve with the help of regulatory effects from Treg cells. In those patients without this specific TCR variant, there is a higher total Ig concentration with no antigen specificity, and through interactions of IL-13 induced polarized M2 macrophages and fibroblasts the granulomatous inflammation can become chronic and progress to permanent fibrotic lesions, regulated by TGF-β and CCL18. The straight solid arrow implies the identified differentiation of T-cell subsets or monocyte/macrophage polarization, while the dotted one indicates the possible conversion of T-cell subgroups. The flexible solid arrow describes the contribution of diverse immune cells to the formation of granuloma and lung fibrosis or granuloma resolution, and the hollow one emphasizes the two significantly different disease outcomes after granuloma formation in sarcoidosis. TLR, toll-like receptor; DCs, dendritic cells; APC, antigen-presenting cell; MHC, major histocompatibility complex; TCR, T cell receptor; TNF, tumor necrosis factor; IFN, interferon; NK: natural killer; IL-2R, interleukin-2 receptor; CXCR, chemokine (C-X-C motif) receptor; CCR, chemokine (C-C motif) receptor; CXCL, chemokine (C-X-C motif) ligand; Treg cells, regulatory T cells; CTLA4, cytotoxic T-lymphocyte antigen 4; TGF, transforming growth factor. Created with BioRender.com.

Despite extensive research over the past several decades, the pathogenesis of sarcoidosis remains incompletely understood. The commonly held view is that the granulomatous process is driven by an exaggerated immune response to the yet unknown antigen (13, 14), including both the adaptive and the innate immune system. This review summarizes recent advances in our understanding of the involved immune cells and their unique roles in disease development and progression (**Figure 1**). It is also aimed to shed new light on directions for future studies and treatment strategies to improve disease outcomes.

## CD4+ T Cells

T cells, especially CD4+ T cells, are key components of sarcoid granuloma. On histochemistry, they are located in the outer layer of the granuloma. Bronchoalveolar lavage (BAL) studies have demonstrated increased numbers of activated CD4+ T cells in the lungs of sarcoidosis patients. They can differentiate into diverse effector T cells depending on differences in the local immune milieus. These effector T cells can secrete various cytokines or chemokines that promote or inhibit the granulomatous inflammation.

### Th1 Cells

An established immunologic feature of sarcoidosis is that the accumulated CD4+ T cells which trigger granuloma formation are strongly Th1 polarized. Early studies demonstrate that lung T lymphocytes from patients with active pulmonary sarcoidosis spontaneously release interleukin (IL)-2 (15) and interferon (IFN)-γ (16), and both lung and blood T lymphocytes in individuals with active pulmonary sarcoidosis spontaneously express functional IL-2 receptors (IL-2R) (17, 18). Both IL-2 and IFN-γ are important Th1 cytokines, implying that there is an exaggerated Th1 immune response involved in the process of sarcoid granuloma formation as shown in bronchoalveolar lavage fluid (BALF) from sarcoidosis patients (19). As demonstrated further, BAL cells from sarcoidosis patients can also release bioactive IL-12 and IL-18 which are important Th1 cytokines and able to synergistically induce IFN-γ production (20). There is a remarkably greater proportion of T cells secreting Th1 type cytokines in BALF than in peripheral blood (21), indicating that the Th1 immune reaction is restricted locally to the lungs. The Th2 cytokine profile (IL-4, IL-5, and IL-10) of lung T cells is decreased instead (22, 23).

Recently, the expression of some chemokines has been shown to be preferentially associated with a Th1 immune response in sarcoid lesions. IFN-γ-inducible protein (IP)-10 or chemokine (C-X-C motif) ligand (CXCL)10, involved in neutrophil and lymphocyte recruitment, is particularly increased in subjects with sarcoidosis (24). The lung accumulation of chemokine (C-X-C motif) receptor (CXCR) 3, chemokine (C-C motif) receptor (CCR) 5, IL-12R and IL-18R expressing T cells is in line with previous reports showing elevated levels of the corresponding ligands in the lungs of sarcoidosis (25). T-bet, the Th1 transcription factor, controls the upregulation of genes for IFN-γ and CXCR3 expression, two crucial molecules in sarcoid inflammation and granuloma formation (26, 27). BAL cells from patients with sarcoidosis express higher levels of T-bet mRNA than those of healthy controls, suggesting a significant role for T-bet in this disease (28). As mentioned above, Th1 cells are indispensable in sarcoidosis.

### Th17 Cells

Th17 cells, characterized by the ability to produce IL-17A, have been extensively studied since their discovery over 10 years ago in many diseases, such as inflammatory bowel disease (IBD) (29), colorectal cancer (30), autoimmune arthritis (31), malignant pleural effusion (32), and hypoxia-induced pulmonary hypertension (33). These studies have revealed the dichotomous nature of Th17 cells, playing a pathogenic role in inflammatory disorders while a protective role in promoting health (barrier protection and pathogen defense) (34).

More recently, the role of Th17 cells in the pathogenesis of sarcoidosis has been recognized. The accumulation of large clonal populations of IL-17A+ CD4+ T cells in blood, BALF and sarcoid tissue surrounding the central core of the granuloma combined with enhanced IL-17A expression in sarcoid tissue indicates the involvement of Th17 cells in granuloma induction and/or maintenance in sarcoidosis (35). Chemokine (C-C motif) ligand (CCL) 20, (the ligand of CCR6), contributes to the recruitment of Th17 cells from the blood into the lungs (36). Th17 cells specific for early secretory antigenic target of 6kD (ESAT-6), a mycobacterial protein, are present in blood and BALF of sarcoidosis patients (37). Mycobacterium tuberculosis catalase-peroxidase (mKatG), another mycobacterial protein, can induce the production of IL-17 in BAL cells from sarcoidosis patients with Löfgren’s syndrome, the acute form of sarcoidosis known to have a particularly good prognosis (38). There is a significant decrease in cytotoxic T-lymphocyte antigen 4 (CTLA4) expression specifically on Th17 cells from mediastinal lymph nodes and BALF in sarcoidosis, contributing to Th17 priming and activation, and resulting in the ongoing active immune response in sarcoidosis (39). Exploring the exact role of Th17 cells in sarcoidosis is a rapidly evolving field of research.

### IFN-γ-Producing Th17 Cells

Although IL-17A production is the hallmark of Th17 cells, this T cell subset expresses many other cytokines or chemokines such as IL-17F, IL-22, IL-26, IFN-γ and CCL20 (40). Under Th17-inducing conditions, human T cells secreting both IL-17 and IFN-γ can arise and display similar functional characteristics to IL-17 single-producing Th17 cells *in vitro* (41). Considerable plasticity in Th17 cells exists. *Ex vivo* isolated Th17 cells can be converted into IFN-γ-producing Th17 cells through combined IFN-γ and IL-12 signaling (42). These cells stably co-express RORγt (the key transcription factor of Th17 cells) (43) and T-bet at the single-cell level, suggesting that they combine the pro-inflammatory potential of Th17 and Th1 cells (42). The nomenclature for this “Th1-polarized Th17 subset” is not uniform. These cells have been referred to as Th17/Th1, Th1/17 or Th17.1 cells. IL-1β, produced by activated macrophages, monocytes and T cells, is the key cytokine that renders pathogen-specific Th17 cells the potential to convert into Th17.1 cells (44, 45). Th17.1 cells may be a group of unclassical Th1 cells, losing the expression of RORγt mRNA and secreting IFN-γ alone through uncertain mechanisms. That’s to be studied in the future.

Accumulating evidence is now indicating that Th17.1 cells play a central role in sarcoidosis. An increased number of Th17.1 cells is present in peripheral blood (37), BALF (46), and mediastinal lymph nodes (47) from sarcoidosis patients. A recent study shows that Th17.1 cells (and not Th1 cells) are the predominant producers of IFN-γ in sarcoidosis BALF, challenging the prevailing hypothesis of the Th1 paradigm in the sarcoidosis pathogenesis (48). The frequency of Th17.1 cells is higher in blood from sarcoidosis patients with pulmonary function test (PFT) impairment, defined by the reduction in absolute FVC or DLCO of 10% or 15%, than in those without, and changes in Th17.1 cells proportion show an inverse relationship with PFT changes during the follow-up (49). Moreover, the proportion of Th17.1 cells in BALF increases with sarcoidosis radiologic stage (46) and closely relates to a chronic disease course (47). To the contrary, another study reports that a higher percentage of Th17.1 cells correlates with a disease phenotype with a more favorable prognosis (50). Further investigation is needed to explore whether the role of Th17.1 cells in the development of sarcoidosis is rather pathogenic or protective.

### Regulatory T (Treg) Cells

Treg cells are important T cell components with strong immunosuppressive capacities on Th cells, B cells, and other immune cells. Treg cells can be separated into different subsets based on the expression of forkhead box P3 (FoxP3), the indispensable transcription factor for their development and function (51). Manipulation of a particular subpopulation, rather than total FoxP3+ cells matters in the functional and numerical analysis of Treg cells. CD25^bright^ FoxP3+ Treg cells pose an immune paradox in sarcoidosis, they exert incomplete inhibition of tumor necrosis factor (TNF) -α production and powerful antiproliferative activity to T cells, leading to the failure in controlling local inflammation and to the abnormal peripheral anergy, respectively (52).

The role of Treg cells in the pathogenesis of sarcoidosis remains controversial. In an *in vitro* model of granuloma formation, peripheral blood mononuclear cells (PBMCs) are cocultured with Bacille Calmette Guerin (BCG) extract-coated beads (53). The depletion of Treg cells in this model accelerates granuloma formation in healthy individuals and in patients with inactive sarcoidosis, while it does not modify granuloma formation in active sarcoidosis patients (53), indicating an impaired suppressive ability of Treg cells in active sarcoidosis. The number of Treg cells is found to be decreased both in blood and BALF from sarcoidosis patients (46, 54–56), with lower expression of FoxP3 in BAL cells (54), and treatment with prednisone induces elevated FoxP3 mRNA levels (55). Further, inhaled vasoactive intestinal peptide (VIP) increases the number of BAL Treg cells *in vivo* and *in vitro* (56). VIP treatment could both convert naive T cells into Treg cells and strengthen their immunosuppressive effects (56). Inversely, there are studies revealing a higher proportion of Treg cells in peripheral blood and BALF but with impaired suppressive capacity (57–59). Restoration of Treg cell function appears to be associated with spontaneous clinical resolution of sarcoidosis (57). Treg cells in BALF from sarcoidosis patients are found to undergo extensive amplifications and highly express IL-4, with impaired repressor activity (58). Increased susceptibility of circulating Treg cells towards CD95L (FAS ligand, FAS-L)-mediated apoptosis is present in sarcoidosis patients, leading to impaired survival of Treg cells (59). An increased percentage of circulating Treg cells at time of diagnosis is seen in patients developing chronic disease during follow-up (59). The expression of miR-34a and miR-146b is higher in Treg cells from BALF with lower expression of miR-150 and miR-223 in comparison with blood, and miR-34a is commonly related to apoptosis (60). To date it remains unclear which precise mechanisms lead to Treg cell dysfunction in sarcoidosis. Less well known is that Treg cells can lose the expression of FoxP3 and express RORγt, instead, thereby turning into Th17 cells in autoimmune arthritis (61). It’s difficult to draw a conclusion whether it suits for sarcoidosis.

## Monocytes/Macrophages

The mononuclear phagocyte system (MPS) consists of a group of bone marrow-derived cells, and includes blood monocytes, diverse tissue macrophages and monocyte-derived dendritic cells, mainly responsible for phagocytosis, cytokine secretion and antigen presentation through the pattern recognition receptor (PRR), such as Toll-like receptor (TLR) 2 (62). In active sarcoidosis, blood monocytes are activated showing increased expression of CD16, CD69, and the integrin very late antigen (VLA)-1 (63). The CD200R/CD200L axis is vital in maintaining immune homeostasis of the lung (64). Blood monocytes from patients with sarcoidosis show reduced expression of CD200R which is linked to enhanced TNF and IL-6 production following PHA stimulation (65). Monocytes are increased not only in blood but also in BALF of sarcoidosis patients. These BAL cells produce TNF without exogenous stimulation and are highest in patients who develop chronic disease (66).

Under inflammatory conditions, blood monocytes are recruited into the affected tissues or organs, where they differentiate into macrophages. Granuloma formation results from a dynamic interplay between macrophages and T lymphocytes. Mature macrophages aggregate, turn into epithelioid and giant cells and form compact granulomas. Alveolar macrophages from sarcoidosis patients secrete large amounts of chemokines such as monokine induced by interferon-γ (Mig)/CXCL9, IP-10/CXCL10, and interferon-inducible T cell α chemoattractant (I-TAC)/CXCL11, which are all CXCR3 ligands and play crucial roles in the accumulation of Th1 lymphocytes in sarcoid lungs (67).

Depending on the microenvironment, macrophages can acquire distinct functional phenotypes, usually referred to as either classically activated macrophages (M1) or alternatively activated macrophages (M2), with pro-inflammatory or anti-inflammatory/profibrotic capacity, respectively (68). Polarized macrophage phenotypes have been described in different diseases/conditions. Dectin-1 induces M1 macrophages in pulmonary paracoccidiodomycosis (69). M1 polarization is involved in obesity and insulin resistance (70). During both the acute and fibrotic phase of bleomycin-induced lung injury, the expression of M2-like macrophage markers is elevated (71), and M2 polarization plays a protective role in the pathogenesis of experimental autoimmune encephalomyelitis (EAE) (72). As for sarcoidosis, discordant findings regarding M1 or M2 polarization have been published as summarized in **Table 1** (73–79).

**Table 1:** Studies on macrophage polarization in sarcoidosis.

Reference	Country	Population	Material	Method	Associated Markers	Result
Wikén M, et al. (73)	Sweden	36 sarcoidosis patients; 17 healthy subjects	Total BAL cells and sorted alveolar macrophages	Quantitative real-time PCR	**M1:** IL-12p35, IL-12p40, IL-23p19, CCL20, CXCL10/11/16, CD80, CD86, CCR7, iNOS	No evidence for alveolar macrophage polarization
					**M2:** IL-10, CCR2, CCL18	
Prokop S, et al. (74)	Germany	7 sarcoidosis patients with lungs and muscle affected; 7 patients with other myopathies containing macrophagocytic infiltration	Muscle biopsies	Immunohistochemistry; Quantitative real-time PCR	**M1:** iNOS, COX2 **M2:** CD206, CD301, SOCS-1, IL-27R, arginase-1	M2 polarized macrophages present in sarcoid granulomas and responsible for myofibrosis in muscle
Preusse C, et al. (75)	France	10 patients with muscular sarcoidosis; 10 patients with macrophagic myofasciitis; 6 patients with subjective fatigability	Skeletal muscle biopsies	Immunofluorescence; Quantitative real-time PCR	**M2:** CD206, MRC1, STAT6, SOCS1	M2 polarization inducing giant cell and typical granuloma formation and fibrogenesis
Honda Y, et al. (76)	Japan	95 consecutive cardiac sarcoidosis patients; 50 patients with nonischemic cardiomyopathy	Endomyocardial biopsies	Immunohistochemistry	**M2:** CD163	M2 macrophages less frequently observed in cardiac sarcoidosis
Wojtan P, et al. (77)	Poland	36 patients with sarcoidosis; 10 HP patients; 8 NSIP patients; 6 IPF patients; 15 patients with other ILD	BAL cells	Immunocytochemistry	**M1:** CD40 **M2:** CD163	A higher proportion of M1 cells in sarcoidosis than in other ILD
Shamaei M, et al. (78)	Iran	10 sarcoidosis patients; 12 tuberculosis patients	Mediastinal lymph nodes and TBLB for sarcoidosis patients; pleural tissue, neck, axillary lymph nodes and TBLB for tuberculosis patients	Immunohistochemistry	**M1:** CD14, CD68 **M2:** CD163	A shift towards M2 macrophage subsets in granulomas from sarcoidosis patients
Locke LW, et al. (79)	USA	20 active sarcoidosis patients (PBMC, lung tissue, mediastinal lymph nodes); 5 volunteers (PBMC) and 6 organ donors (lung tissue and mediastinal lymph nodes)	PBMC incubated with PPD-coated polystyrene beads; lung and mediastinal lymph node tissues	*In vitro* human sarcoidosis model; Immunofluorescence; ELISA	**M1:** IFN-γ **M2:** IL-10, IL-13, CD163	Strong M2 polarization in sarcoidosis

BAL, bronchoalveolar lavage; PCR, polymerase chain reaction; HP, hypersensitivity pneumonitis; NSIP, nonspecific interstitial pneumonia; IPF, idiopathic pulmonary fibrosis; ILD, interstitial lung disease; TBLB, transbronchial lung biopsy; PBMC, peripheral blood mononuclear cell; PPD, purified protein derivative; ELISA, enzyme-linked immunosorbent assay.

Early studies indicate that there is no evidence for a M1 or M2 polarization in sarcoidosis patients when comparing the relative gene expression of M1 or M2 associated markers in total BAL cells or sorted alveolar macrophages (73). More recent work, however, show a higher proportion of M1 alveolar macrophages in pulmonary sarcoidosis patients, expressing more CD40 and less CD163 (cell surface markers for M1 and M2, respectively) than in other interstitial lung diseases (ILDs) (77). Likewise, CD163+ M2 macrophages have less frequently been observed in cardiac sarcoidosis patients (76). In contrast, the number of CD163+ macrophages in mediastinal lymph node and transbronchial lung biopsy specimens is increased in sarcoidosis patients compared with tuberculosis patients and correlates with the radiologic stages, indicating a shift towards M2 polarization in sarcoidosis in advanced stages (78). Also in muscular sarcoidosis a strong Th2-M2 polarization and a significant expression of transforming growth factor β (TGF-β) or CCL18 is found in macrophages and considered to be important for granuloma formation and myo-fibrosis development (74, 75). Moreover, as shown in an *in vitro* human sarcoidosis model, IL-13-mediated M2 polarization participates in early granuloma formation (79). Taken together, the functional and phenotypic diversity of macrophages in sarcoidosis is evident from these studies. Current evidence suggests the transition from a M1 to a dominant M2 phenotype in more advanced stages of sarcoidosis (74, 75, 78). The precise role of M2 polarization in the development of chronic disease and fibrosis is not well defined and should be further explored.

## Other Immune Cells

Collections of B cells are located in the outer layer of the granulomas, and B-cell activating factor (BAFF) levels are increased in serum of sarcoidosis patients and related to disease activity and severity (80). High frequencies of somatic hypermutations in IgA and IgG transcripts, with normal serum immunoglobulin levels, are observed in sarcoidosis patients, who reach remission spontaneously through the recognition with specific TCR Vα2.3/Vβ22, inducing T-B-cell interaction (81). The anti-CD20 monoclonal antibody rituximab shows a therapeutic effect in sarcoidosis (82, 83). On the other hand, rituximab can induce a sarcoid-like reaction when patients with refractory pemphigus vulgaris (PV) are treated with this drug (84). Recent studies have identified the expansion of diverse B cell subpopulations, a regulatory phenotype, so-called B regulatory (Breg) cells (85, 86), and a novel subset named age-associated B cells (ABCs), in patients with sarcoidosis (87). Taken together, these results support a role of B cells in the pathogenesis of sarcoidosis.

Dendritic cells (DCs) are distributed throughout the body and are the professional antigen-presenting cells (APCs) of the immune system. Although most researches have focused on alveolar macrophages as the primary pathogenic APCs in sarcoidosis, emerging evidence indicates that DCs play a crucial role (88). While total numbers of DCs and myeloid DCs (mDCs) are decreased in blood (89, 90), there are increased numbers of mDCs in BALF (91, 92) from sarcoidosis patients, inducing T cell proliferation and differentiation. In patients with sarcoidosis, the expression of markers for mature DCs such as CD83 and CD86 on mDCs is decreased in BALF (93), while large numbers of mature DCs are found in granuloma-containing biopsies (90, 91), indicating an abnormal DCs maturation status within different compartments of the lung.

Natural killer (NK) cells are able to secrete a broad panel of pro- and anti-inflammatory cytokines, displaying different phenotypes and functional activities. In sarcoidosis patients, a particular subset, the CD56^bright^ NK cell population, capable of producing substantial cytokines, is more frequent in BALF than in blood (94). Upon stimulation with PMA and ionomycin *in vitro*, there is an increase of IFN-γ and TNF-α producing CD56^bright^ NK cells in BALF from sarcoidosis patients (94), which may imply involvement of NK cells in granuloma formation. On the other hand, the percentage of NK cells in BALF from sarcoidosis patients is lower than in other ILDs, including hypersensitivity pneumonitis (HP) (95). However, another study found no difference in the proportion of NK cells in BALF between sarcoidosis and HP patients (96). Increased numbers of NK cells in BALF from sarcoidosis patients have been found to be associated with a poor outcome and a higher probability to require corticosteroid treatment (97). There is a lack of consensus on the role of NK cells in sarcoidosis which calls for further investigations.

Natural killer T (NKT) cells, a unique subgroup of lymphocytes bearing surface markers of both NK cells and T lymphocytes, bridge innate immunity and adaptive immunity. Compared with HP patients, patients with sarcoidosis have lower frequency of BAL NKT cells (95, 96), with the frequency tending to be higher in Löfgren’s syndrome (96). There are two major subsets of NKT cells, CD1d-dependent cells (also called Vα24 invariant NKT cells, iNKT cells for short) with immunoregulatory properties and CD1d-independent cells. Complete loss or striking reduction of iNKT cells in peripheral blood and absence in mediastinal lymph nodes and granuloma tissue occurs in sarcoidosis patients, suggesting that the loss of immunoregulatory effects by iNKT cells contributes to the amplification and persistence of the T cell immune response (98). Reduced iNKT cell numbers in blood may be responsible for defective IL-10 production and T cell suppression by monocytes, leading to the exaggerated immune response in sarcoidosis (99). Moreover, reduced circulating iNKT cell numbers are found to be associated with signs of pulmonary fibrosis on CT scans and other clinical indicators of disease severity or activity, such as reduced FVC and increased C-reactive protein (CRP) (100). Both the single IFN-γ producing iNKT cells and the dual functional IFN-γ and TNF-α producing iNKT cells are decreased in blood (99). In accordance with the results in blood, there is a striking reduction of iNKT cells in BALF from sarcoidosis patients with no difference in clinical phenotypes, negatively correlating with increased CD4+ T cells in BALF (101). Interestingly, this concordance of the blood and BALF compartment with respect to reduced iNKT-cell numbers is unusual and needs further exploration since most immune response features are diminished in blood and enhanced in BALF of sarcoidosis patients.

## Concluding Remarks

A series of consecutive studies spanning several decades delineate a critical role for various immune cells in the immunopathogenesis of sarcoidosis. Recent work has identified the contribution of diverse subsets of CD4+ T cells in this disease, with emphasis on the role of Th17 cells, Th17.1 cells and Treg cells as effector cells involved in the formation or resolution of sarcoid granuloma. In parallel, emerging evidence points to macrophages and M2 polarization that may induce the transition to fibrosis in advanced disease stage. The role of B cells, DCs, NK cells, and NKT cells is also outlined in this review. Though great progress has been made in the understanding of sarcoidosis, further work remains to be done for unravelling the precise mechanisms and immunopathology underlying the disorder. Studies with meticulous databases of phenotypically well-defined patients are needed, equipped with samples, from both baseline and follow-up investigations, of peripheral blood, BALF, lung, mediastinal lymph nodes, and other affected tissues. Hopefully, such research will answer important clinical questions such as why certain patients will experience spontaneous resolution, others will keep persistent low grade inflammatory activity, and a minority will progress to irreversible fibrosis.

## Author Contributions

HZ took part in decision on structure and content of the review, performing literature, search, and writing the review. UC revised the draft critically and gave final approval of the submitted version. HD took part in the revision of the draft and gave thorough feedback throughout the process, and accepted the final version. All authors contributed to the article and approved the submitted version.

## Funding

This study was supported by National Natural Science Foundation of China [No. 81870056 & 92068108].

## Conflict of Interest

The authors declare that the research was conducted in the absence of any commercial or financial relationships that could be construed as a potential conflict of interest.

## Publisher’s Note

All claims expressed in this article are solely those of the authors and do not necessarily represent those of their affiliated organizations, or those of the publisher, the editors and the reviewers. Any product that may be evaluated in this article, or claim that may be made by its manufacturer, is not guaranteed or endorsed by the publisher.
